# Supplementary figures and images for: Deciphering the 3D Structural Characterization of Gonadotropin-Releasing Hormone in Tenualosa ilisha Using Homology Modeling, Molecular Dynamics, and Docking Approaches
Source: Int J Mol Sci. 2025 Jun 25;26(13):6098. doi: 10.3390/ijms26136098 (PMC12249859; doi:10.3390/ijms26136098)

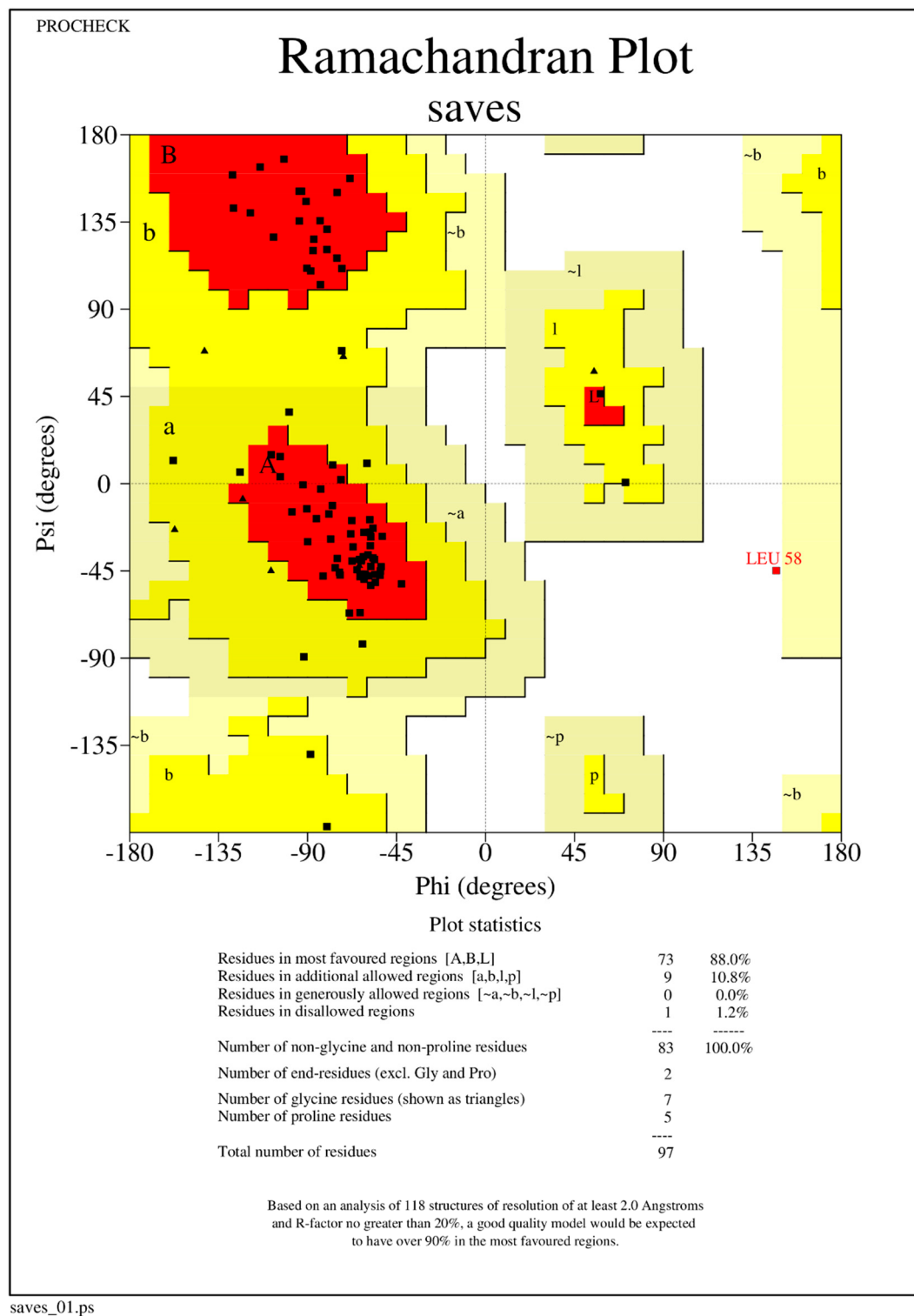

**Supplementary figure S1:** Ramachandran plot of the GnRH-1 stereochemical quality

Supplement: Supplementary file 1 [file ijms-26-06098-s001.zip › ijms-3654012-supplementary.pdf]
